# A new Oriental Agalliini leafhopper genus (Hemiptera, Cicadellidae, Megophthalminae), with description of one new species and three new combinations

**DOI:** 10.3897/zookeys.1296.206709

**Published:** 2026-10-30

**Authors:** Bin Zhang, Juntong Wang, M. D. Webb

**Affiliations:** 1 College of Life Sciences & Technology, Inner Mongolia Normal University, Hohhot 010022, China College of Life Sciences & Technology, Inner Mongolia Normal University Hohhot China; 2 Key Laboratory of Biodiversity conservation and Sustainable utilization for College and University of Inner Mongolia Autonomous Region, Hohhot 010022, China Key Laboratory of Biodiversity conservation and Sustainable utilization for College and University of Inner Mongolia Autonomous Region Hohhot China; 3 Lord Wandsworth College, Long Sutton, Hook, RG29 1TA, Hampshire, UK Lord Wandsworth College Long Sutton United Kingdom; 4 Department of Science (Insects), the Natural History Museum, London SW7 5BD, UK Department of Science (Insects), the Natural History Museum London United Kingdom

**Keywords:** Auchenorrhyncha, morphology, new genus, taxonomy

## Abstract

A new Agalliini leafhopper genus, *Orientagallia* Zhang & Webb, **gen. nov**. (Cicadellidae, Megophthalminae), is described to place four Oriental species: *O. viraktamathi* Zhang & Webb, **sp. nov**. from Yunnan Province, China; *O. priyankae* (Viraktamath), **comb. nov**. from China (new record), India, and Nepal; *O. kolasibensis* (Meshram), **comb. nov**. and *O. shillongensis* (Meshram), **comb. nov**. both from India. A checklist to the species of *Orientagallia*, **gen. nov**. and a key for their identification are provided.

## Introduction

The leafhopper tribe Agalliini (Cicadellidae: Megophthalminae) comprises rather homogeneous wedge-shaped leafhoppers distributed in all geographical regions. In the Oriental region, their host plants include seven monocotyledons in one family and 41 dicotyledons in 19 families (Viraktamath 2011). In recent years, several taxonomic revisions of the tribe Agalliini have been conducted based on material taken elsewhere in the Oriental region (Viraktamath 2011), China (Viraktamath et al. 2012), and Madagascar (Viraktamath and Gonçalves 2013).

Material from Yunnan, China, has recently revealed a new species similar in general habitus and male genital morphology to *Igerna kolasibensis* Meshram, *I. priyankae* Viraktamath, and *I. shillongensis* Meshram, from the Indian subcontinent. However this new species belongs to a separate generic lineage from *Igerna*. Based on detailed morphological comparisons, a new genus, *Orientagallia* Zhang & Webb, gen. nov. is herein established to accommodate the new species and three previously described species.

## Material and methods

Terminology used follows Oman (1949) and Dietrich (2005), but terminology of female genitalia is according to Davis (1975) and the leg chaetotaxy is based on Rakitov (1997). Techniques for the preparation of genital structures follow Oman (1949). Images were produced with Leica M205 C. Holotypes and paratypes are deposited in the College of Life Sciences and Technology, Inner Mongolia Normal University (**IMNU**).

## Taxonomy

### Orientagallia

Taxon classificationAnimaliaHemipteraCicadellidae

Zhang & Webb gen. nov.

C6E104CF-FE4B-51FB-B2AE-880B689206EA

https://zoobank.org/314BA435-FA4F-4EC8-AB2E-FDE4BD3A4C3C

#### Type species.

*Orientagallia viraktamathi* Zhang & Webb, sp. nov., designated here.

#### Diagnosis.

This is a genus similar to *Dianagallia* Zhang & Webb, 2026 (Zhang et al. 2026) in having paired black spots on the vertex, pronotum, and antennal sockets, a strongly sclerotized anal collar bearing elongate process, and a T-shaped connective with the lateral arms shorter than the stem. It is distinguished from *Dianagallia* by the male genitalia: aedeagus with distinct preatrium extending below apex of connective; shaft without processes or with ventral subapical process (in *Dianagallia*, aedeagus with pair of lamellate apical processes and preatrium absent); pygofer without processes (in *Dianagallia*, pygofer with single long, spine-like process on each side).

#### Description.

***Colouration***. Colour ochraceous with dark-brown to black markings on head and thorax. Vertex with two large, round black spots, closer to each other than to eyes; antennal sockets dark brown; posterior half of pronotum with two subtriangular black markings.

***Morphology***. Head with posterior margin sinuately curved laterally behind eyes. Ocelli closer to eyes than to each other. Forewing with three anteapical and four apical cells present; inner anteapical cell open posteriorly. Hind tibial macrosetae arranged as PD 8–12, AD 7–8, AV 6–7. Hind basitarsus with one platella in distal transverse row.

***Male genitalia***. Pygofer trapezoidal in lateral view, with a dorsocaudal lobe and narrow dorsal bridge. Anal collar present with each lateral branch bearing a well-developed, elongate apical process. Subgenital plates lacking long, stout setae. Styles with inner arm either as long as or longer than outer arm. Connective T-shaped, with lateral arms shorter than stem. Aedeagus simple; shaft tubular or laterally compressed, curved dorsad; gonopore apical on ventral surface; preatrium present, short, extended below apex of connective stem; dorsal apodeme well developed, laterally compressed, with or without a basal ventral process.

***Female genitalia***. Valvulae as in Fig. 3j–m. First valvulae, in lateral view, with alveolate sculpture in distal two-thirds. Second valvulae, in lateral view, with distinct dorsal hyaline and sclerotized area.

#### Distribution.

Oriental region (China, India).

#### Etymology.

The generic name is derived from the combination of “Orienta” for Oriental (the distribution of the new genus) and the suffix -*agallia*, referring to the type genus of Agalliini. Gender feminine.

#### Remarks.

The new genus runs to *Austroagallia* Evans in the last couplet of the key to Oriental Agalliini (Viraktamath 2011), but it can be readily distinguished from that genus in having the ocelli closer to the eyes than to each other (rather than distinctly closer to each other), aedeagus symmetrical (as opposed to strongly asymmetrical), and connective with stem longer than lateral arms (rather than stem and lateral arms always nearly equal in length). *Orientagallia* also shares several diagnostic features with *Dianagallia* Zhang & Webb, including a pair of black spots on the vertex, pronotum, and antennal sockets; a strongly sclerotized anal collar bearing and elongate process; and a T-shaped connective with the lateral arms shorter than the stem. However, *Orientagallia* can be readily distinguished from *Dianagallia* in having the aedeagus with a distinct preatrium lying below the apex of the connective and shaft with or without a ventral subapical process (in *Dianagallia*, aedeagus has a pair of lamellate apical processes and the preatrium is absent); the pygofer in *Orientagallia* lacks processes (in *Dianagallia*, pygofer bears a single long, spine-like process). *Orientagallia* is also similar to *Igerna* but differs in having the ocelli closer to the eyes than to each other (in *Igerna* sensu stricto, ocelli are slightly closer to each other than to the eyes), well-developed and strongly sclerotized anal collar with an elongate process that is bifurcate or apically laterally expanded (in *Igerna* the anal collar is simple or reduced), a T-shaped connective with the lateral arms distinctly shorter than the stem (in *Igerna*, the lateral arms are subequal in length to the stem), and the aedeagal base not sunken into the dorsal apodeme and the shaft dorsally curved (in *Igerna*, aedeagal base is usually sunken into the dorsal apodeme and the shaft is typically curved ventrad).

##### Checklist to species of *Orientagallia* gen. nov.

*O. kolasibensis* (Meshram, 2015): 242 comb. nov.—India (Mizoram)

*O. priyankae* (Viraktamath, 2011): 72–73 comb. nov.—India (Meghalaya), Nepal (Kathmandu), and China (Yunnan, new record)

*O. shillongensis* (Meshram, 2015): 243 comb. nov.—India (Meghalaya), China (Yunnan)

*O. viraktamathi* Zhang & Webb, sp. nov.—China (Yunnan)

##### Key to species of *Orientagallia* gen. nov.

**Table:** 

1	Anal collar process with apex laterally expanded, spatulate	***O. kolasibensis***
–	Anal collar process with forked apex (Fig. 3b)	**2**
2	Aedeagal shaft tubular, without apical processes; ventral margin of dorsal apodeme bearing a single process (Fig. 3f)	***O. viraktamathi* sp. nov**.
–	Aedeagal shaft laterally compressed, with or without a short ventral process near apex	**3**
3	Aedeagal shaft slightly wider basally than apically in lateral view with an apical ventral process (Fig. 4f)	***O. privankae***
–	Aedeagal shaft distinctly wider basally than apically without an apical ventral process	***O. shillongensis***

### Orientagallia viraktamathi

Taxon classificationAnimaliaHemipteraCicadellidae

Zhang & Webb sp. nov.

606A55A5-1413-5374-B0F5-A352F2877B73

https://zoobank.org/96C00CD5-0308-4007-9653-499F865E76BC

Figs 1a–d, 2a, b, 3

#### Diagnosis.

This species is similar to *O. kolasibensis* but can be distinguished by the following: aedeagal shaft less strongly sinuate, with apex non-bifurcate, and hind margin of female seventh sternite slightly truncated.

**Figure 1.**
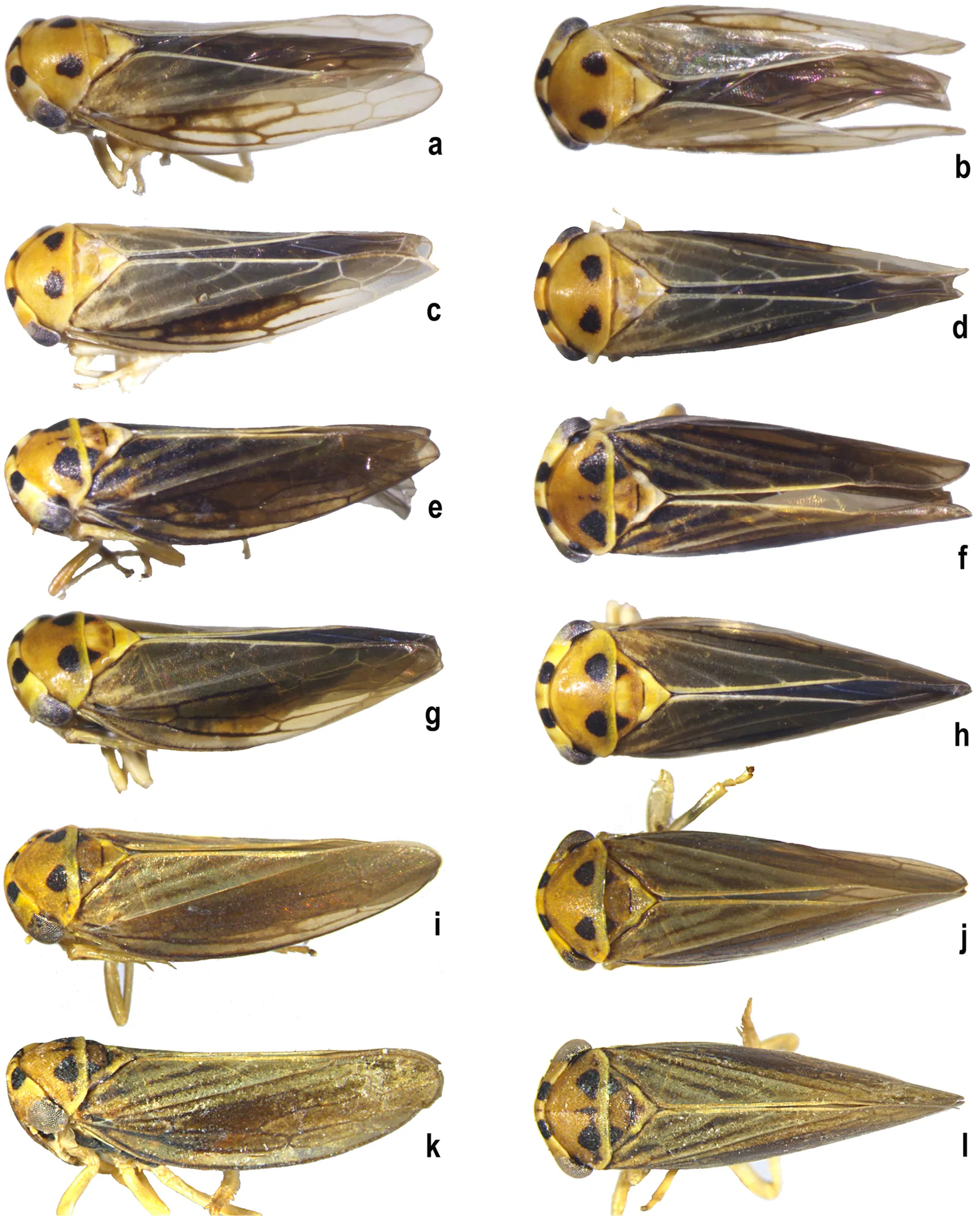
*Orientagallia* species, lateral and dorsal habitus, male. **a–d**. *O. viraktamathi*; **e–h**. *O. privankae* (Dehong & Lincang); **i–l**. *O. priyankae* (Baoshan & Yuxi) (**a, c, e, g, i, k**. Lateral habitus; **b, d, f, h, j, l**. Dorsal habitus; **a, b, e, f, i, j**. Male; **c, d, g, h, k, l**. Female).

**Figure 2.**
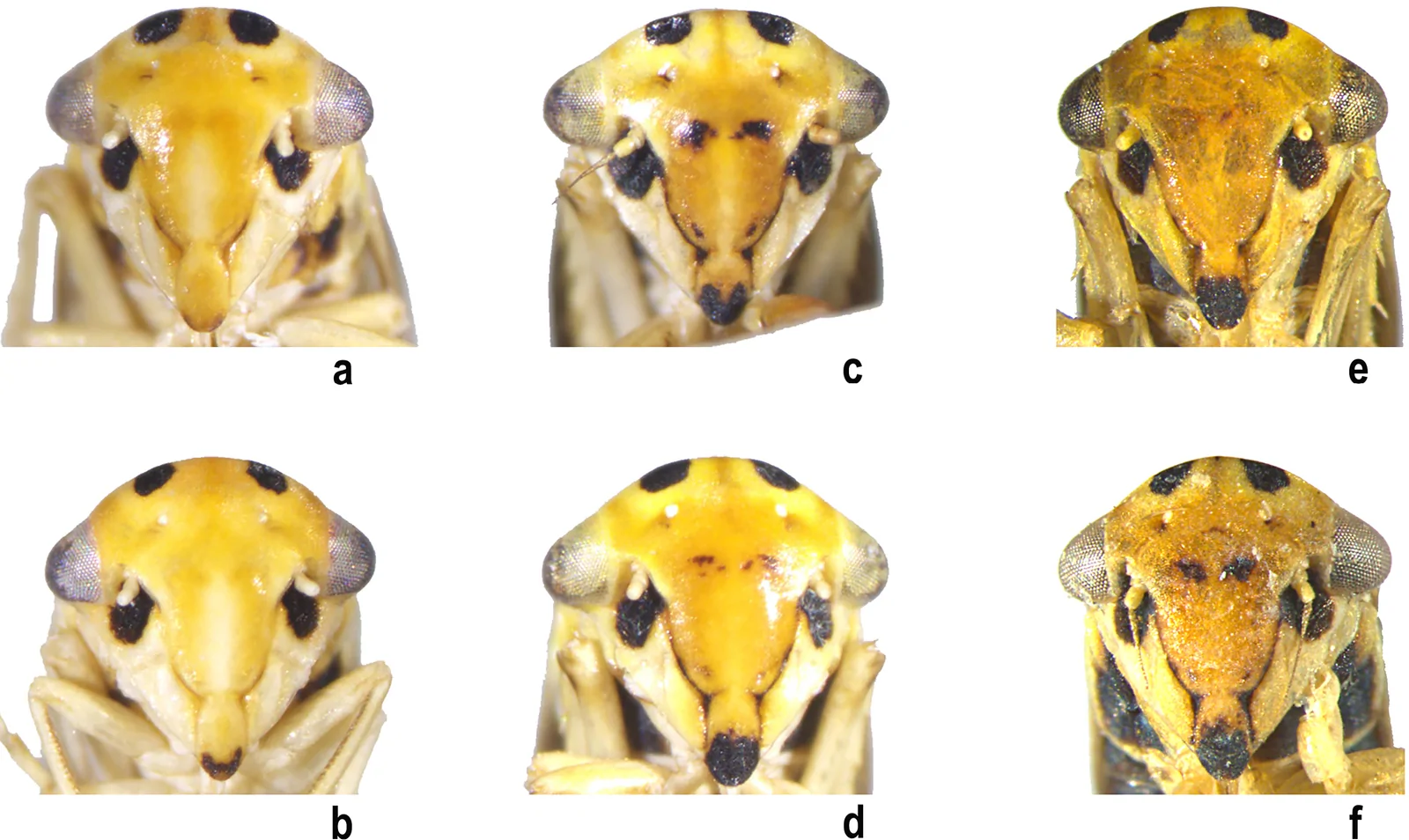
Facial aspect of *Orientagallia* species. **a, b**. *O. viraktamathi*; **c, d**. *O. privankae* (Dehong and Lincang); **e, f**. *O. priyankae* (Baoshan and Yuxi) (**a, c, e**. Male; **b, d, f**. Female).

**Figure 3.**
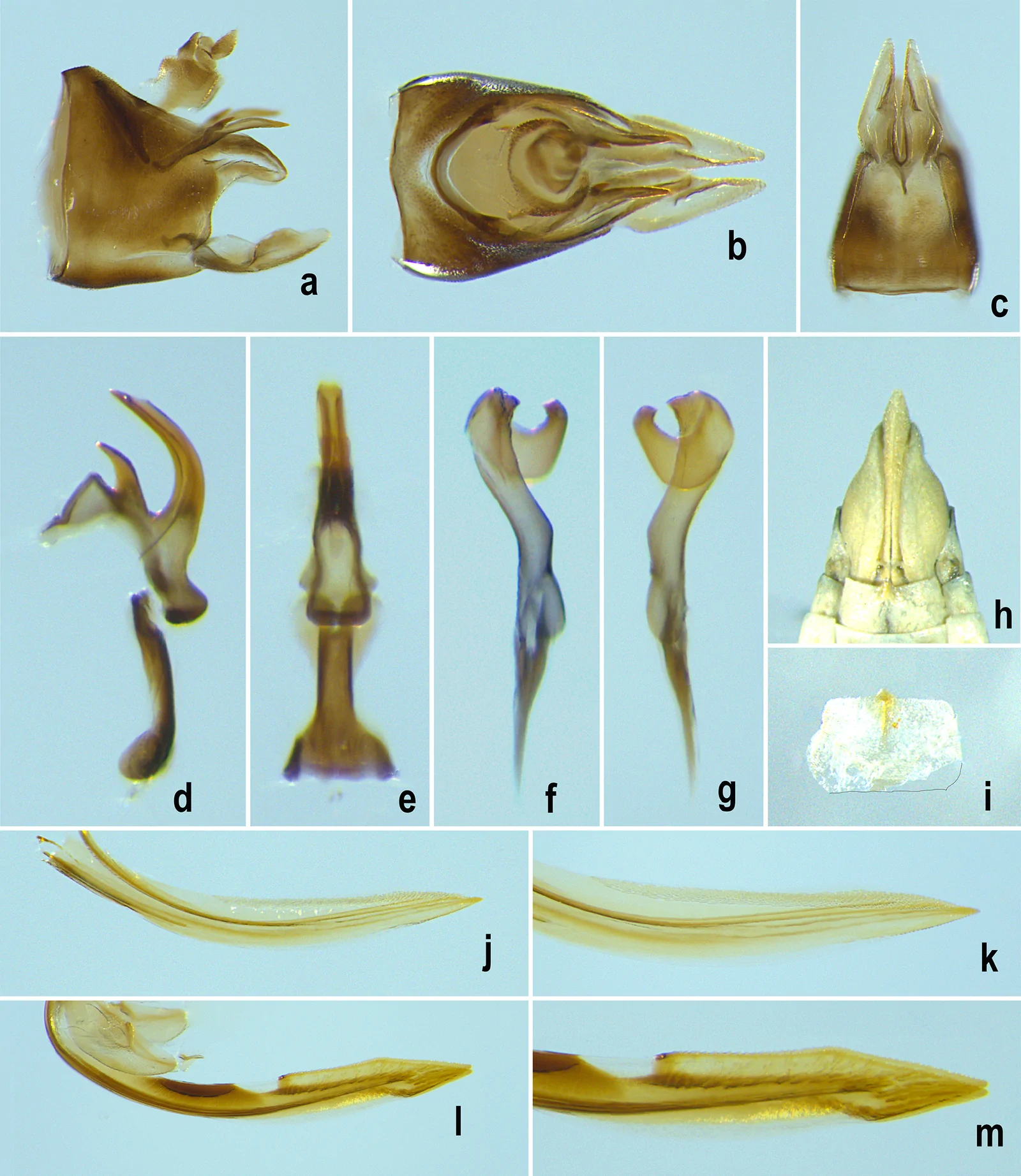
*Orientagallia viraktamathi* Zhang & Webb, sp. nov. **a**. Male genital capsule, lateral view; **b**. Ibid., dorsal view; **c**. Subgenital plates; **d**. Aedeagus and connective, lateral view; **e**. Ibid., ventral view; **f, g**. Style, lateral view; **h**. Apex of abdomen; **i**. Seventh sternite; **j, k**. First valvulae; **l, m**. Second valvulae.

#### Description.

**Male**. Face (Fig. 2a) pale yellow to yellowish brown; apical lateral margins of frontoclypeus and apical margins of clypellus dark brown; small transverse dark-brown macula below ocelli. Mesoscutum (Fig. 1b) with pair of yellowish-brown maculae on laterobasal portions. Forewings (Fig. 2a, b) pale greyish brown with greyish-white veins.

***Male genitalia***. Pygofer (Fig. 3a) trapezoidal in lateral view, with dorsal margin evenly concave and produced posteriorly into finger-like dorsocaudal lobe. Anal collar process (Fig. 3a, b) with dorsal fork much shorter (about one-fifth length of ventral fork). Styles (Fig. 3d, e) with inner arm stout; outer arm short, bearing sharp tooth at apex. Connective (Fig. 3f, g) with stem slightly shorter than aedeagal shaft, nearly straight in lateral view, and with lateral margins subparallel in ventral view. Aedeagus (Fig. 3f, g) with shaft broad at base, gradually narrowing towards apex, strongly curved dorsad, apically obliquely truncated and nearly parallel to dorsal apodeme and its process; dorsal apodeme well developed, bearing short process arising from mid-ventral margin and slightly curved dorsad, process about one-third length of shaft; preatrium slightly shorter than dorsal apodeme, about two-thirds its length.

**Female**. Similar in overall colouration to male, but transverse macula (Fig. 2d) below ocelli indistinct, and dark brown marking on apex of anteclypeus larger and arcuate.

***Female genitalia***. Seventh sternite (Fig. 3h, i) with posterior margin slightly concave and median triangular lobe with acute apex.

#### Measurements.

Male: body length 3.88–4.07 mm. Female: body length 4.25–4.46 mm.

#### Material examined.

***Holotype***: • ♂, China: Yunnan Province, Lincang, Cangyuan County, Nangunhe National Nature Reserve, 1282 m, 3. vi. 2021, collected by B. Zhang; ***Paratypes***: • 7♂♂, 8♀♀, same date as holotype; • 8♂♂, 11♀♀, China: Yunnan Province, Lincang, Cangyuan County, 2072 m, 6. vi. 2021, collected by B. Zhang; • 18♂♂, 23♀♀, China: Yunnan Province, Lincang, Cangyuan County, Nangunhe National Nature Reserve, 1510 m, 8. vi. 2021, collected by B. Zhang; • 14♂♂, 17♀♀, China: Yunnan Province, Lincang, Cangyuan County, 1378 m, 12. vi. 2021, collected by B. Zhang; • 2♂♂, 2♀♀, China: Yunnan Province, Dehong, Yingjiang County, 295 m, 13. vi. 2025, collected by B. Zhang.

#### Etymology.

The species is named in honour of Prof. C.A. Viraktamath, a systematichemipterist, in recognition of his outstanding contributions to taxonomy of the Megophthalminae.

### Orientagallia priyankae

Taxon classificationAnimaliaHemipteraCicadellidae

(Viraktamath) comb. nov.

0611FAD8-458A-5691-875E-F7B8B8B6F63F

**Figure 4.**
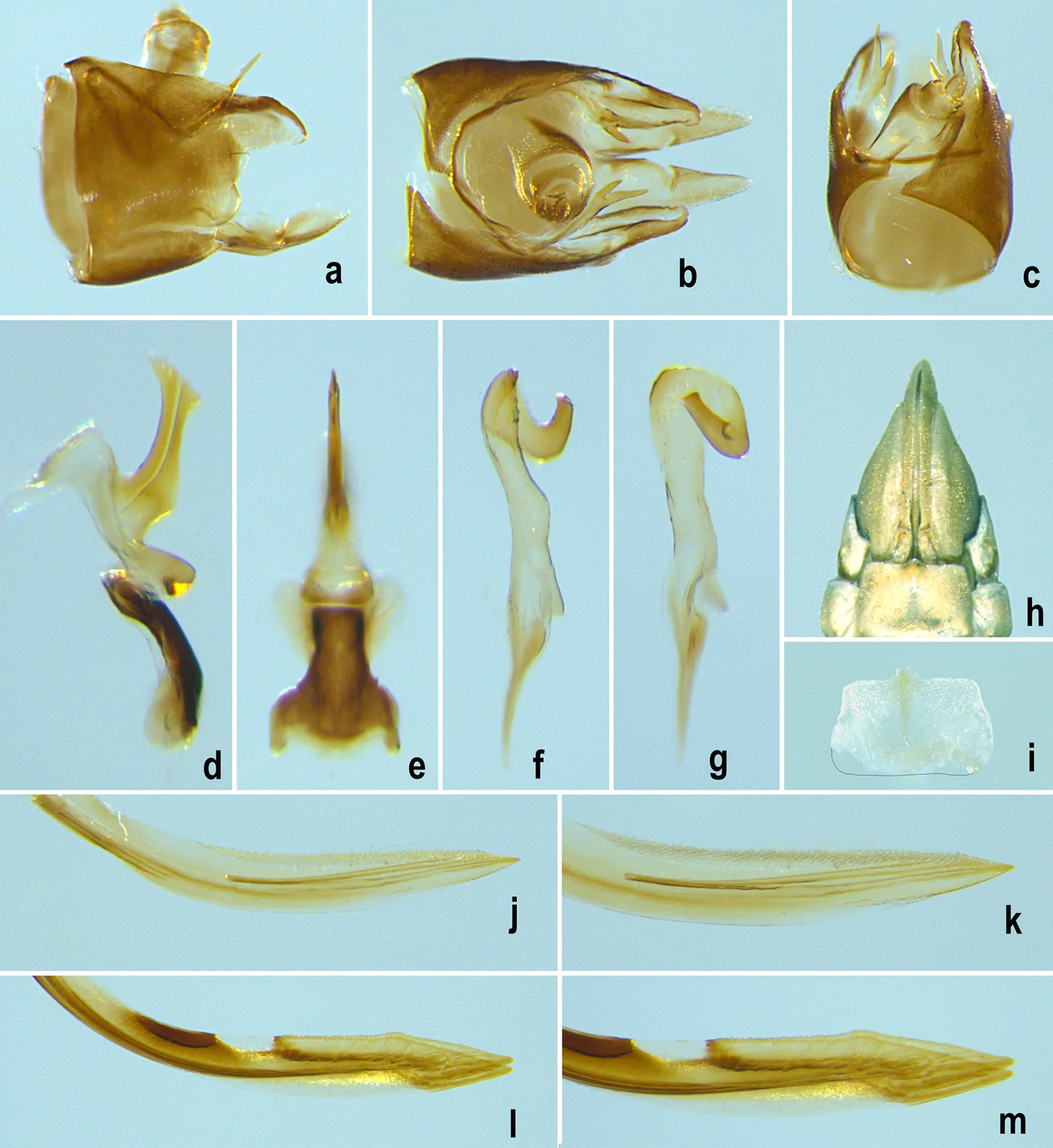
*Orientagallia privankae* (Viraktamath) (Dehong and Lincang) comb. nov. **a**. Male genital capsule, lateral view; **b**. Ibid., dorsal view; **c**. Subgenital plates; **d**. Aedeagus and connective, lateral view; **e**. Ibid., ventral view; **f, g**. Style, lateral view; **h**. Apex of abdomen; **i**. Seventh sternite; **j, k**. First valvulae; **l, m**. Second valvulae.

**Figure 5.**
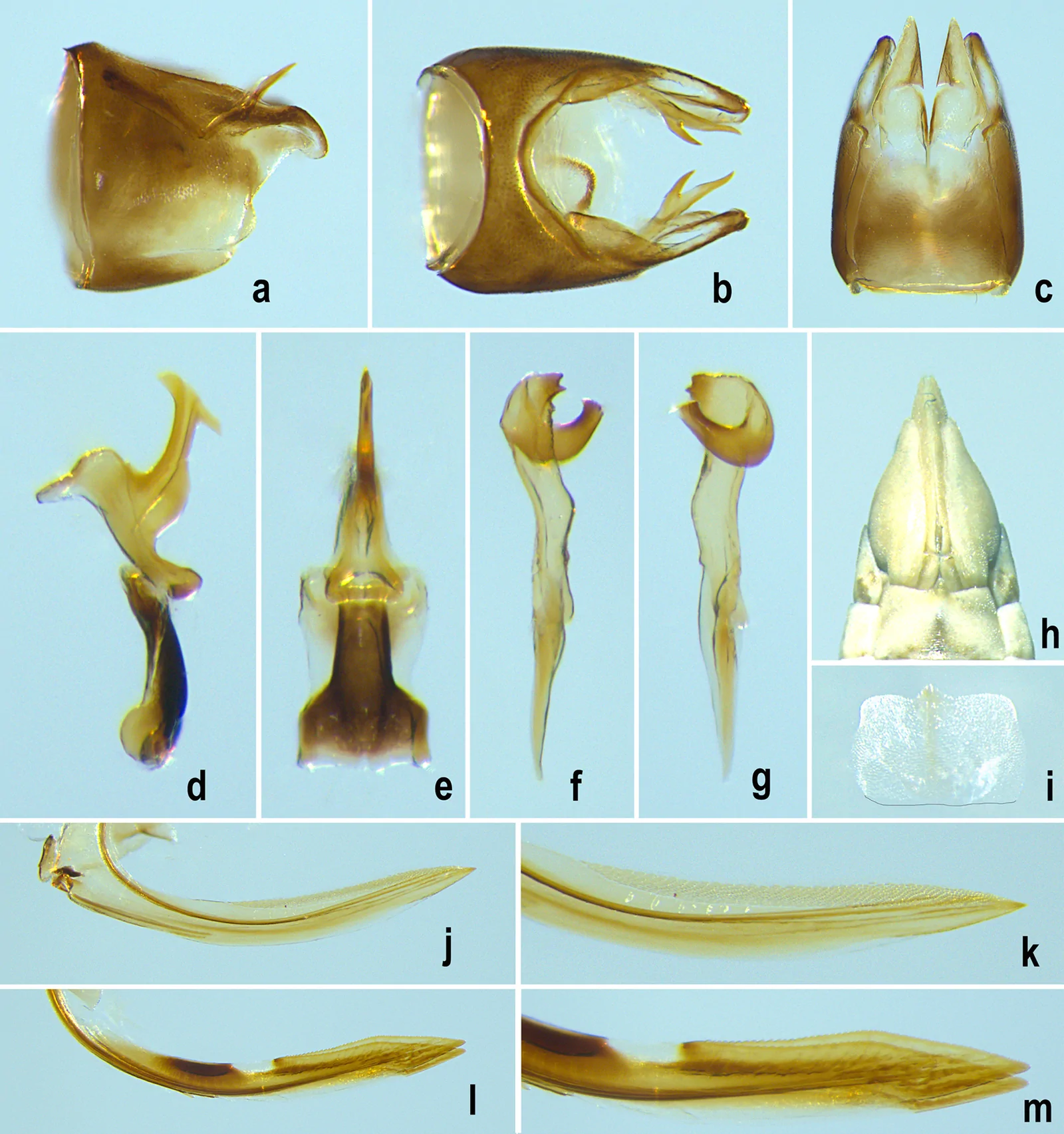
*Orientagallia priyankae* (Viraktamath) (Baoshan and Yuxi) comb. nov. **a**. Male genital capsule, lateral view; **b**. Ibid., dorsal view; **c**. Subgenital plates; **d**. Aedeagus and connective, lateral view; **e**. Ibid., ventral view; **f, g**. Style, lateral view; **h**. Apex of abdomen; **i**. Seventh sternite; **j, k**. First valvulae; **l, m**. Second valvulae.

Figs 1e–l, 2c–f, 2c, d, 4, 5

Igerna priyankae Viraktamath, 2011: 72, figs 343–350; Meshram et al. 2015: 249, figs 37–44.

#### Description.

Male pygofer (Fig. 5a) trapezoidal; dorsal margin nearly straight from base to apex, then slightly concave before dorsocaudal lobe; dorsocaudal lobe with posterodorsal margin obliquely straight. Anal collar process (Fig. 5b) with dorsal fork equal to or less than half length of ventral fork. Styles (Fig. 5d, e) with inner arm short; outer arm with apex bearing two teeth. Connective (Fig. 5f, g) with stem broad basally, tapering towards apex and slightly curved dorsad subapically. Aedeagus (Fig. 5f, g) with shaft laterally compressed, basally expanded into a rounded protuberance and apically expanded dorsoventrally into a lamellate process; ventral process narrow and blade-like, slightly longer than bluntly rounded dorsal process; dorsal apodeme subquadrate, slightly shorter than preatrium, with anterodorsal corner produced into a long, finger-like process.

#### Measurements.

Male: body length 3.69–3.81 mm. Female: body length 4.06–5.9 mm.

#### Material examined.

• China, Yunnan Province: 2♂♂, 4♀♀, Baoshan, Mt. Gaoligongshan, 1875 m, 28. iv. 2009, collected by B. Zhang; • 5♂♂, 6♀♀, Baoshan, Mt. Gaoligongshan, 1856 m, 7. v. 2010, collected by B. Zhang; • 8♂♂, 10♀♀, Baoshan, Mt. Gaoligongshan, 1890 m, 13. vi. 2011, collected by B. Zhang; • 2♂♂, 5♀♀, Pianma County, 18. vi. 2011, collected by B. Zhang; • 6♂♂, 8♀♀, Yuxi, Xinping County, Mt. Ailaoshan, 1206 m, 2. v. 2022, collected by W. Wang; • 5♂♂, 7♀♀, Mt. Gaoligongshan, 1880 m, 2. v. 2023, collected by J.T. Wang; • 2♂, 3♀♀, Dehong, Yingjiang County, 1318 m, 3. vi. 2011, collected by B. Zhang; • 4♂♂, 5♀♀, Dehong, Yingjiang County, 1324 m, 12. vi. 2025, collected by B. Zhang; • 2♂♂, 4♀♀, Dehong, Yingjiang County, 295 m, 13. vi. 2025, collected by B. Zhang; • 5♂♂, 8♀♀, Dehong, Yingjiang County, 1603 m, 13. vi. 2025, collected by B. Zhang; • 14♂♂, 17♀♀, Lincang, Cangyuan County, 1378 m, 12. vi. 2021, collected by B. Zhang.

#### Remarks.

This species was previously recorded from Nepal and India, and we record it here from China for the first time. This species can be readily distinguished from other species by the laterally compressed aedeagal shaft with an apical process and an apical ventral process. There is some variation in the specimens we figure (compare Figs 4, 5) and those previously figured (Viraktamath 2011; Meshram et al. 2015) in the length and shape of the aedeagal shaft and its processes and number of apical teeth on the outer arm of the style. These differences are considered to be variation within a single species, pending further studies.

### Orientagallia kolasibensis

Taxon classificationAnimaliaHemipteraCicadellidae

(Meshram) comb. nov.

9849F91B-773A-5256-855C-8219FD8D2A27

Igerna kolasibensis Meshram in Meshram et al. 2015: 242, figs 1–4, 9–16, 27–31.

#### Description.

Lateral margins of the frontoclypeus with irregular dark-brown longitudinal stripes; clypellus dark brown except at base. Anal collar process with apex laterally expanded and spatulate. Aedeagus similar to that of *O. viraktamathi*, but with a shorter shaft and dorsal apodeme lacking a short process.

#### Remarks.

This species was originally described from Mizoram, India. No material was examined in the present study; however, it has been adequately described and illustrated in the original description.

### Orientagallia shillongensis

Taxon classificationAnimaliaHemipteraCicadellidae

(Meshram) comb. nov.

3817B290-5B9F-5BF3-BFAD-5422E8F2B85F

Igerna shillongensis Meshram in Meshram et al. 2015: 243, figs 5–8, 17–26, 32–36; Zhan et al. 2018: 61, figs 1d–f, 3a–d, 5a–d.

#### Description.

Pronotum with a longitudinal stripe between a pair of triangular, dark-brown markings. Anal collar process with dorsal fork approximately one-third length of ventral fork. Aedeagus small, shaft broad at base; dorsal apodeme short and poorly developed.

#### Remarks.

This species was described from Meghalaya, northeastern India, adjacent to China. No material of this species was examined in the present study, but it was adequately described and illustrated in the original description and recorded from Dehong, Yingjiang County, Yunnan Province, China by Zhan et al. (2018).

## Supplementary Material

XML Treatment for Orientagallia

XML Treatment for Orientagallia viraktamathi

XML Treatment for Orientagallia priyankae

XML Treatment for Orientagallia kolasibensis

XML Treatment for Orientagallia shillongensis

## Additional information

### Artificial Intelligence (AI) use

The authors accept full responsibility for the content of the manuscript, including the disclosure of any use of AI.

No AI tools were used in the preparation of this manuscript.

### Author ORCIDs

B. Zhang https://orcid.org/0000-0001-8702-9022

J. Wang https://orcid.org/0009-0003-4902-9760

M.D. Webb https://orcid.org/0000-0002-1312-6142

### Data availability

All of the data that support the findings of this study are available in the main text.
